# Reconstruction of Moderately Constricted Ears by Combining V-Y Advancement of Helical Root, Conchal Cartilage Graft, and Mastoid Hitch

**Published:** 2016-07-08

**Authors:** Ahmed Elshahat, Riham Lashin

**Affiliations:** Plastic Surgery Department, Faculty of Medicine, Ain Shams University, Cairo, Egypt

**Keywords:** congenital constricted ears, cartilage grafts, mastoid hitch, lop ear, cup ear

## Abstract

**Objective:** Despite the multitude of corrective procedures described in the literature, adequate surgical correction of the congenital constricted ear remains a challenge. The maintenance of the shape and size of the reconstructed upper neohelix poses a particular problem. **Methods:** In the present study, a total of 12 cases of reconstruction were undertaken. All of them were moderate (type IIA Tanzer classification) deformities. A combined procedure was adopted using a V-Y advancement of the helical root, cartilage scoring, and cartilage grafting from the contralateral concha to reconstruct the upper helix. A mastoid hitch was used as an adjunct to these procedures to maintain helical elevation and prevent recurrence. Mean follow-up period was 6 months. **Results:** Results were excellent (*n* = 7), good (*n* = 4), and fair (*n* = 1). Paired *t* test showed a significant increase in the height of the constricted ear postoperatively (*P* < .001) and a nonsignificant difference between the height of the constricted and contralateral ears postoperatively (*P* > .05). Apart from dislodgment of the mastoid hitch suture in 1 patient, no complications were recorded. **Conclusion:** This combined technique is useful in correcting moderately constricted ear deformities.

Congenital ear deformities range from very mild deformities as accessory auricle deformity to severe deformity as microtia. Constricted ear deformity is one of these congenital anomalies. Its severe form is the microtia. Davis^1^ defined the constricted ear as a syndrome affecting all elements of the upper third of the ear in various degrees, whereby it looks like as if the rim of the ear has been tightened by a purse string. Tanzer^2^ coined the term “constricted ear” to minimize the confusion of a multitude of descriptive terms, including “lop,” “cup,” “lidded,” “canoe,” and “cockleshell ears.” This simple term of constricted ear combines the elements of the overhanging upper pole (lop) with the degree of protrusion (cup) to describe a single abnormality with a spectrum of variety. There are 4 main components to this abnormality.^3^ Lidding, which is caused by reduced or absent fossa triangularis, scapha, and superior crus that contribute to the flattened and overhanging helical rim. Diminished support to the upper pole by an abnormal auricularis superior muscle facilitates collapse.^1^ Flattened antihelix and helical rim deepen the conchal fossa, resulting in protrusion. Decreased ear size is a main component caused by the deformities of the fossa triangularis, scapha, and superior crus. Low ear position is rarely found in a moderately constricted ear deformity.

In 1975, Tanzer^2^ classified constricted ears into 3 groups and 2 subgroups according to severity and the reconstructive procedure required. Group I: helical collapse only; group II: deficiency of the scapha, superior crus, and fossa triangularis; group IIa: no supplemental skin needed to expand the auricular margin; group IIb: supplemental skin necessary to expand the auricular margin; and group III: attachment of the anterior helix close to the lobule, the auricle is pouch-like, and the ear is usually low-set.

More than 20 different surgical procedures have been described for the correction of this abnormality.^4^ However, none of these procedures are superior.^5^ When the helical rim cartilage is slightly deformed, reinforcement can be done by a conchal cartilage graft^6^ or a costal cartilage strip from the first or second floating ribs.^7^ When both the helix and the scapha are involved, the whole plastic surgical bag of tricks is emptied for this group: Banner flaps,^2^^,^^3^^,^^8^ V-Y advancement,^3^^,^^9^ or Z advancement^10^ of the root of helix, expanding the cartilage by splitting it into interdigitating leaves,^2^^,^^3^^,^^8^^,^^11^ conchal cartilage grafts,^2^^,^^6^^,^^12^ T-bar costal cartilage graft,^13^ and addition of local skin flaps.^2^^,^^3^^,^^14^ All techniques aim to elongate the upper pole.

A mastoid hitch, whereby the refashioned upper neohelix is sutured to the mastoid fascia, is often used as an adjunct to these procedures to maintain helical elevation and prevent recurrence.^8^^,^^10^ Groups IIB and III of Tanzer are needed to be placed in a different context and regarded as conchal type microtia and corrected accordingly.^13^

To our knowledge and after revising the literature, there were no previous studies that combined the use of V-Y advancement of the root of helix together with conchal cartilage graft and mastoid hitch as a routine procedure to correct moderately constricted ears. The aim of this study was to investigate the reliability and reproducibility of combining these techniques together to treat the moderately constricted ear deformity.

## PATIENTS AND METHODS

### Patients

This study included a total of 12 Tanzer-type IIA constricted ears in 12 patients. All the constricted ears were reconstructed by the senior author in 2015.

Patient age at operation was variable; 9 children whose age ranged from 6 to 10 years (mean age = 8.1 ± 1.5 years), and 3 adults whose age ranged from 27 to 55 years (mean age = 42.3 ± 14 years). There were 2 female patients and 10 male patients. All cases were unilateral. The contralateral ears included 2 normal ears and 10 protruded ears. Seven protruded ears were only set back, and 3 underwent the creation of antihelical folds in addition.

The distribution of the age, gender, uni/bilateral, Tanzer type, contralateral ear deformity, and the procedures done on the contralateral ear in each patient is shown in Table 1. The vertical height of the constricted and contralateral ears pre- and postoperatively in each patient, the size difference of the constricted ear pre- and postoperatively in each patient, the size difference of the contralateral ear pre- and postoperatively in each patient, and the size differences between the constricted and contralateral ears pre- and postoperative in each patient were measured. Ear height was measured from the uppermost point of the helix to the lowermost point of the lobule.

A previously published scoring system for clinical results after ear reconstruction was used.^15^ According to the scoring system, the grade is excellent if the patient has good symmetry in size and position, nice helical rim, good antihelical fold, lobule in correct position, and natural look. The grade is good if the patient has nice shape but deficient in one of the previous details. The grade is fair if the patient is pleased but surgeon is unhappy with the outcome. The grade is poor if problems with size and details may be attributable to underlying deformity or scarring, or if some improvement followed the surgery but lacked details, overall not satisfactory size or position, or both. Photography were taken from behind, with the head slightly tilted downward, from front, with the head tilted upward, to show the size difference and symmetry, and a lateral view, to show the shape of the ear.

### Methods

All procedures were performed under general anesthesia. V-Y advancement of the helical root was used at the upper pole of the ear to release the constricted ear and to add skin to this part. Limited postauricular incisions were made to create the antihelix and to set back the ears using the conchoscaphal (Mustarde suture) and perform the mastoid hitch. Anterior scoring of the cartilage at the zone of antihelix was done routinely in all of our cases. Conchal cartilage grafts were harvested using the anterior approach in cases with well-formed antihelix of the contralateral ear or through the posterior approach in cases where creation of the antihelix is required. Conchomastoid suture of the contralateral ear was used in all cases through the anterior or posterior approach. Mastoid hitch was then used to maintain the height and shape of the neohelix.^8^ No drains were used in any of our cases. And the skin was redraped and closed with 6-0 Proline sutures. Vaseline gauze and cotton dressing were used to maintain the auricular profile postoperative. A firm but comfortable head bandage was applied and removed after 1 week.

### Statistical methodology

Data analysis was done by IBM computer using SPSS (Statistical Program for Social Science, version 16) as follows:

- Description of quantitative variables as mean, SD, and range.
- Description of qualitative variables as number and percentage.
- Paired *t* test was used to compare quantitative variable in the same group pre- and postoperatively:
  ○ *P* > .05 insignificant.
  ○ *P* < .05 significant.
  ○ *P* < .001 highly significant.^16^

## RESULTS

Peri- and postoperative periods were uneventful. Mean follow-up period was 6 months. Follow-up photographs were taken at 1 week, 1 month, and 6 months.

All V-Y flaps survived. There were no infections or hematomas, no delayed wound healing or wound dehiscence, and no skin necrosis. The scar was hidden within the helical rim and hardly visible, and no keloid or hypertrophic scars had developed. Donor site morbidity was negligent.

All patients were satisfied with the outcome of the operation because of the general form, the upright position of the upper pole of the ear, and restoration of symmetry. Revision of mastoid hitch was required in 1 patient.

The results were rated according to the level of involvement.^15^ There were 7 excellent, 4 good, 1 fair, and no poor results; the only patient with fair results underwent revision under local anesthesia. This patient was an adult patient who experienced collapse to the upper helix and the upper part of the ear was slightly protruding compared with the contralateral side, which was easily corrected with revision of the mastoid hitch to maintain the neohelix in the elevated position. The result after revision of the mastoid hitch was good, and the patient was satisfied with the outcome.

Analyzing the vertical height of the constricted and contralateral ears and the size difference between both ears pre- and postoperatively shows statistically significant difference between both ears preoperatively, while the difference was nonsignificant postoperatively by using the paired *t* test, which means that we approached symmetry between both ears in all of our patients as shown in Table 2.

The mean preoperative vertical height of the constricted ears was 5.1 ± 0.4, whereas the mean postoperative vertical height of the constricted ears was 5.8 ± 0.3. The vertical height of the constricted ear increased postoperatively, with a statistically highly significant increase in the vertical height by using the paired *t* test as shown in Table 3.

The mean preoperative vertical height of the contralateral ears was 5.7 ± 0.4, whereas the mean postoperative vertical height of the contralateral ears was 5.9 ± 0.4. The vertical height of the contralateral ear increased postoperatively, with a highly statistically significant increase in the vertical height by using the paired *t* test as shown in Table 4.

The mean change in the vertical height of the constricted ears (5.9 ± 1) was significantly higher than the mean change in the vertical height of the contralateral ears (1.9 ± 0.5) as shown in Table 5. Pre- and postoperative photographs of the 5 patients are shown in Figures 1-22.

## DISCUSSION

Approaching the symmetry in cases of the unilateral constricted ear is an elusive goal, and a combination of different procedures is needed. That is why a combination of V-Y advancement of the helical root, conchal cartilage graft, and mastoid hitch procedures was performed in the current study.

The number of patients in the current study was reasonable statistically and comparable with that of the previous study by Horlock et al,^8^ whose study was conducted on 19 constricted ears in 17 patients, and was greater than that in another study by Kon and Van Wijk,^13^ who used 9 ears in 8 patients and only 4 patients in another study by Al-Qattan.^17^

Patient age in the current study was variable. The study included 9 children, whose age ranged from 6 to 10 years (mean age = 8.1 ± 1.5 years), and 3 adults, whose age ranged from 27 to 55 years (mean age = 42.3 ± 14 years), in comparison with the previous study, which included only young age group patients (age range from 5 to 13 years; mean age = 7.4 years) by Horlock et al^8^ and from 6 to 12 years in another study by Al-Qattan.^17^ On the contrary in the study conducted by Kon and Van Wijk,^13^ the mean age of patients was much older; it ranged from 12 to 36 years (mean age = 19.5 years).

V-Y advancement of the root of helix was used to reconstruct the constricted ear deformity in studies conducted by Cosman^3^ and Stenstrom,^9^ whereas conchal cartilage grafting was used in other studies by Tanzer,^2^ Park,^6^ and Paredes et al.^12^ There were no recorded studies that combined both techniques together. On the contrary, Horlock et al^8^ added the mastoid hitch as an adjunctive to other techniques as V-Y advancement in some cases. The current study standardized the use of all these modalities (V-Y helical root advancement, conchal cartilage grafting, and mastoid hitch) together in all cases.

The present study depended on the ear measurements by comparing the vertical height of both ears pre- and postoperatively and the clinical assessment as described by Chana et al.^15^ The same assessment tools were used in previous study by Horlock et al.^8^ Other studies depended on the general form, the upright position of the upper pole of the ear, and restoration of symmetry.^13^^,^^17^ Therefore, this current study is more objective.

A limited postauricular incision rather than a lateral incision^11^ described in many cup ear reconstructions was used in this study to reduce scar visibility. Lateral incision was not used in this study because the final scar after unfolding of the constricted helix may end up very anteriorly. Another reason was that the pocket created by V-Y advancement would optimally cover the conchal cartilage graft. A third cause was that V-Y advancement together with the limited postauricular incision was enough to release the constricted helix without the need for any additional incisions.

Anterior scoring of the cartilage was used to refashion the angulated cartilage leaf and create the antihelical folds. V-Y advancement of the anterior helical root similar to the procedure described by Stenstrom^9^ provided good cartilage access and a very acceptable scaring. It was more useful in release of the upper helix constriction and allowed more pocket for the cartilage graft. This procedure released the tight anterior structures, which might have contributed to recurrence of the deformity. V-Y advancement in addition contributed to the elevation of the helical rim.

Stephenson^18^ and Kon and Van Wijk^13^ used costal cartilage grafts with a small donor scar and minimal postoperative inconvenience. But, still, there was a donor site scarring. Therefore, Musgrave^19^ modified this technique by using conchal cartilage rather than costal cartilage. The same was advocated in the current study to avoid donor site morbidity of the costal cartilage graft.

To obtain greater expansion (for more severe deformities), the double banner flaps have been used.^2^ But it weakens the cartilage framework of the upper helix and leads to recurrence of the deformity.^8^ Therefore, in the current study, V-Y advancement of the root of helix was used to expand the angulated cartilage of the upper helix, and conchal cartilage graft was used to maintain this expansion.

Importation of the additional skin is needed for type II deformities by a variety of techniques.^14^^,^^20^^,^^21^ In the current study, importation of the additional skin was not a requirement for type IIA constricted ears: first, because of V-Y advancement of the root of helix released the tightness of the skin anteriorly, and, second, because of the dissection of the postauricular pocket over the mastoid fascia released the skin posteriorly and facilitated tension-free closure as a result of the elastic properties of the undermined skin.

The described technique in the present study was suitable for only type IIA deformities. Because type IIB and III Tanzer deformities need to be placed in a different context and regarded as concha-type microtia and corrected accordingly.

## CONCLUSION

This study adopted a simple procedure to correct 12 cases of constricted ears classified as moderate deformities (type IIA) according to the criteria of Tanzer. We concluded that these deformities are best corrected by a V-Y advancement of the helical root, cartilage scoring, conchal cartilage grafts from the contralateral ear, and a mastoid hitch to maintain helical shape, height, and position and to prevent recurrence. This combined technique was reproducible and reliable in reconstruction of moderate-degree constricted ear deformities.

## Figures and Tables

**Figure 1:**
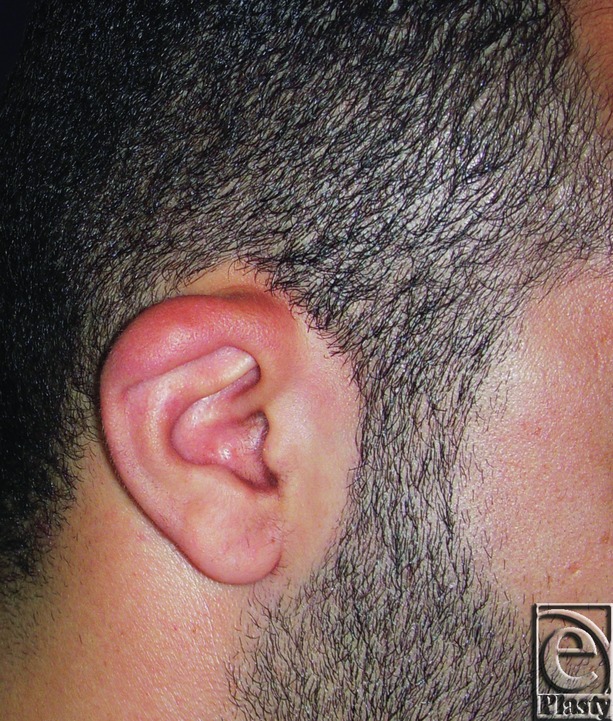
Constricted right ear in a 27-year-old male patient.

**Figure 2:**
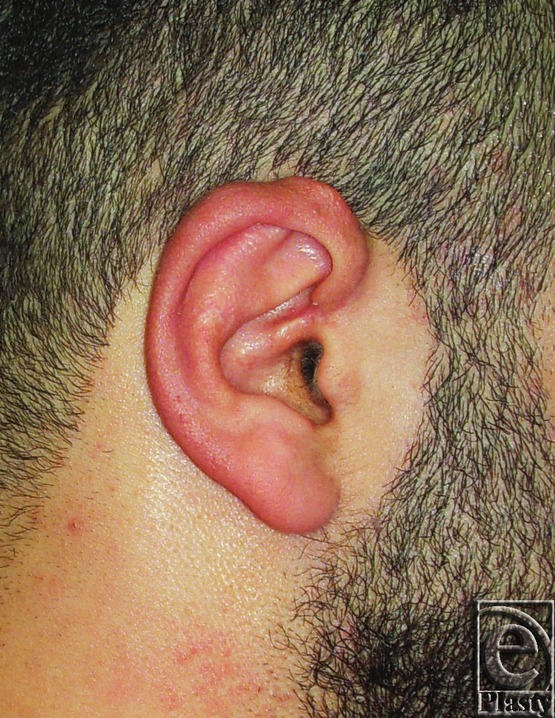
Postoperative photograph for the same ear in Figure 1.

**Figure 3:**
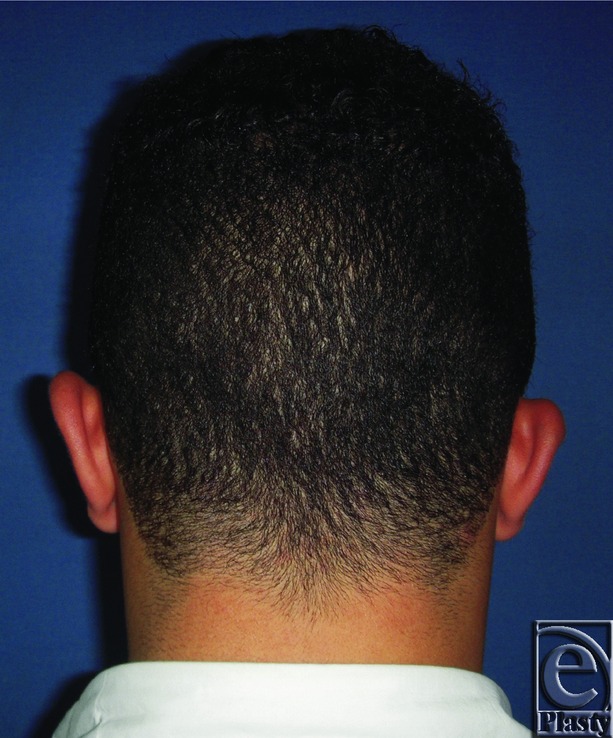
Preoperative posterior view of both ears in a 27-year-old male patient.

**Figure 4:**
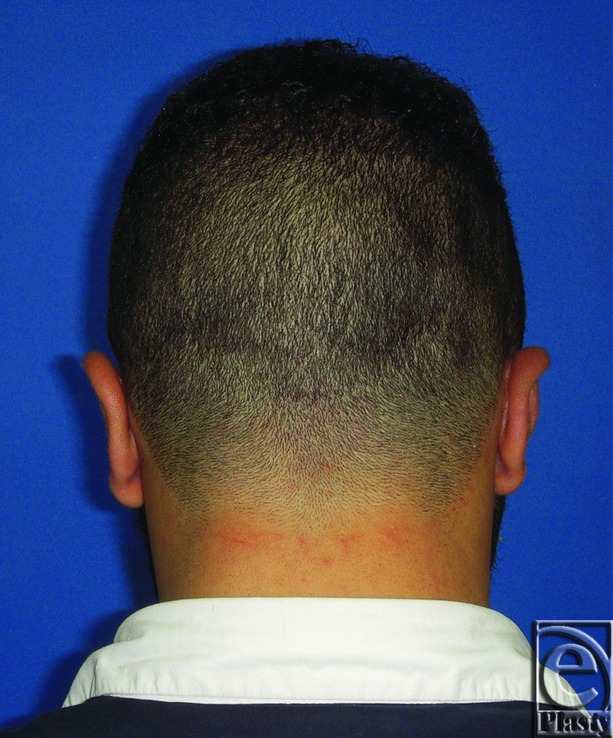
Postoperative posterior view of both ears for the same patient in Figure 3.

**Figure 5:**
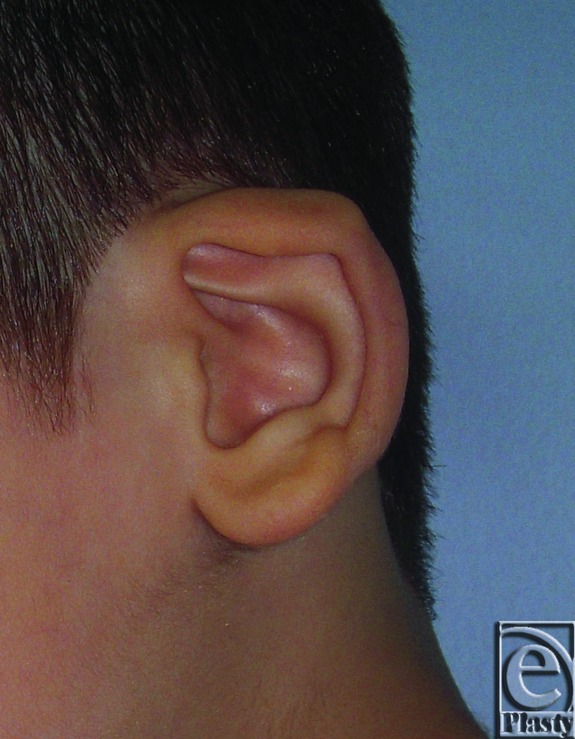
Constricted left ear in an 8-year-old male patient.

**Figure 6:**
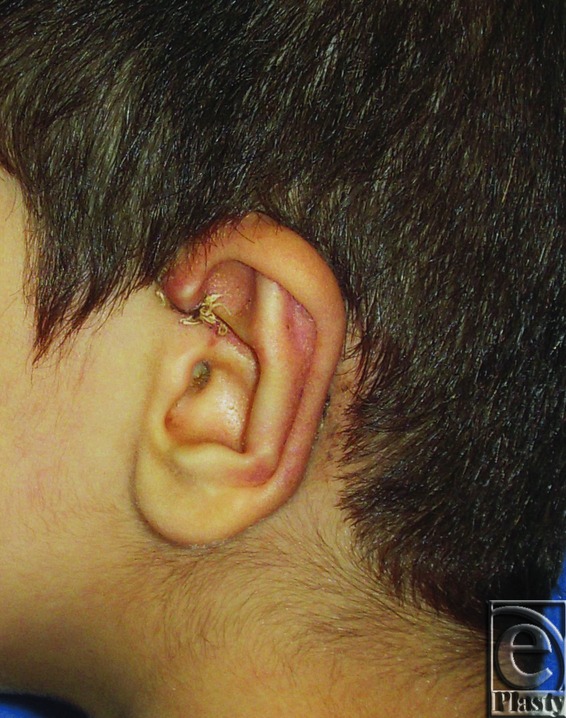
Postoperative photograph for the same ear in Figure 5.

**Figure 7:**
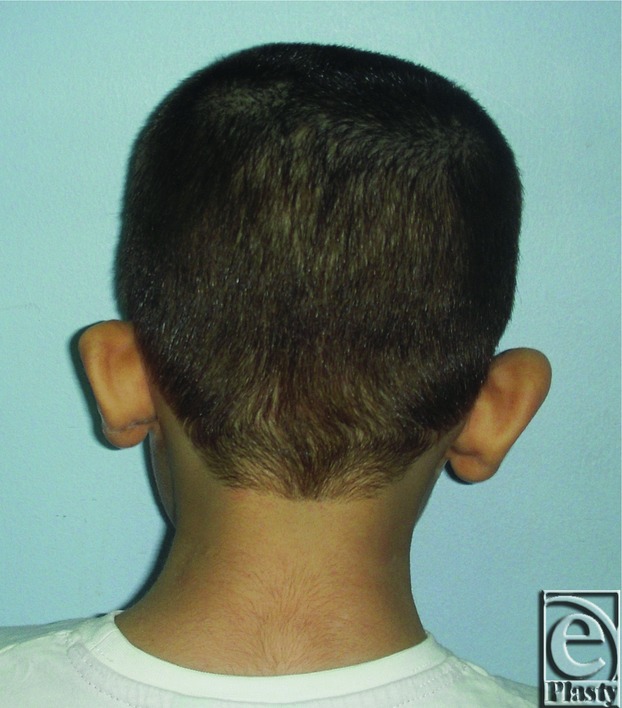
Preoperative posterior view of both ears in an 8-year-old male patient.

**Figure 8:**
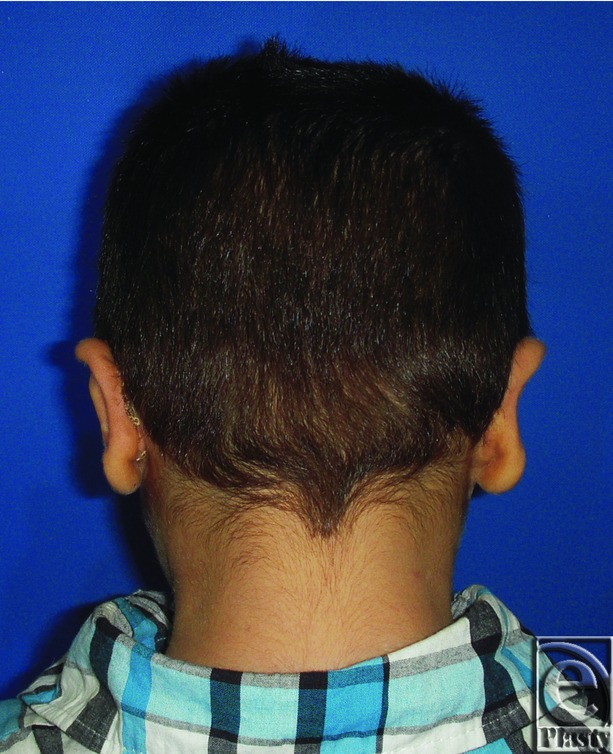
Postoperative posterior view of both ears for the same patient in Figure 7.

**Figure 9:**
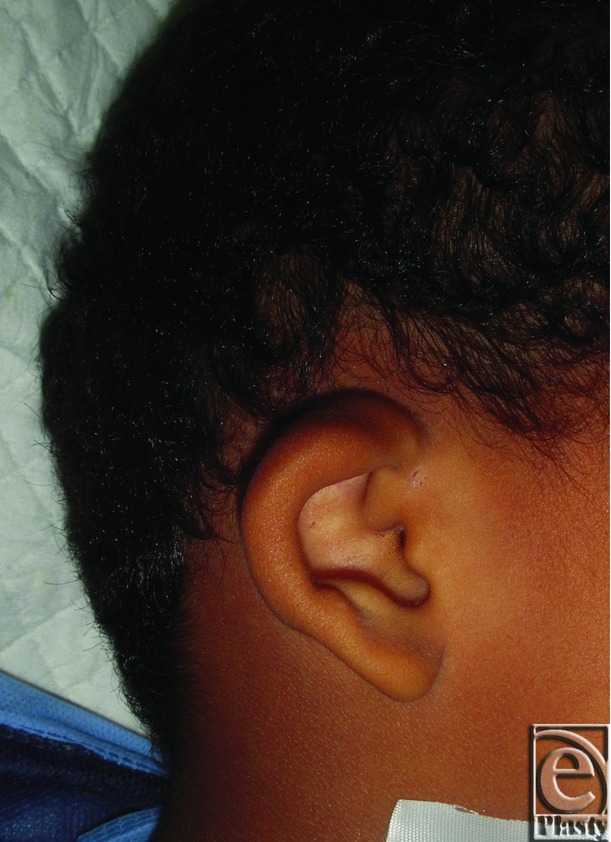
Constricted right ear in a 7-year-old male patient.

**Figure 10:**
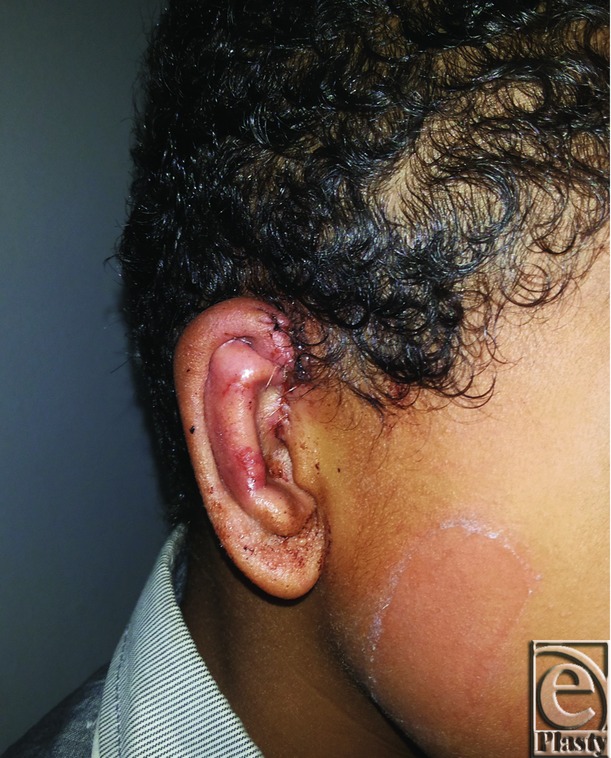
Postoperative photograph for the same ear in Figure 9.

**Figure 11:**
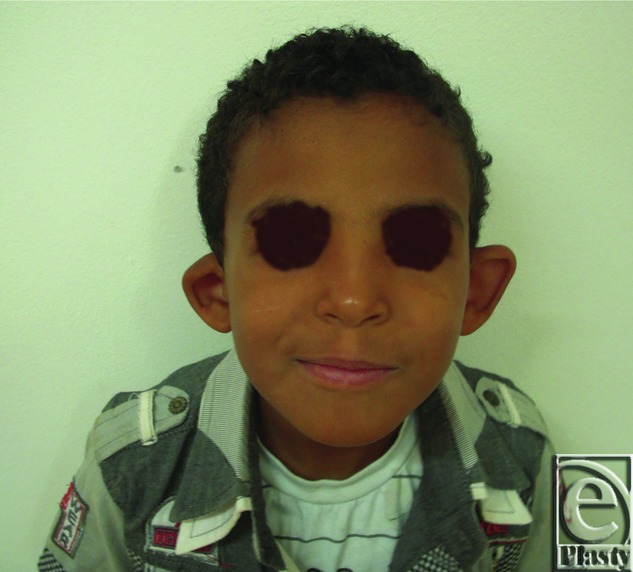
Preoperative anterior view of both ears in a 7-year-old male patient.

**Figure 12:**
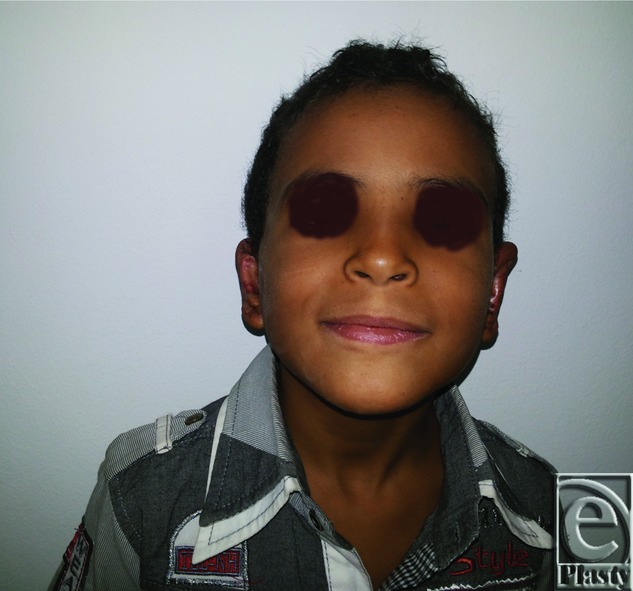
Postoperative anterior view of both ears for the same patient in Figure 11.

**Figure 13:**
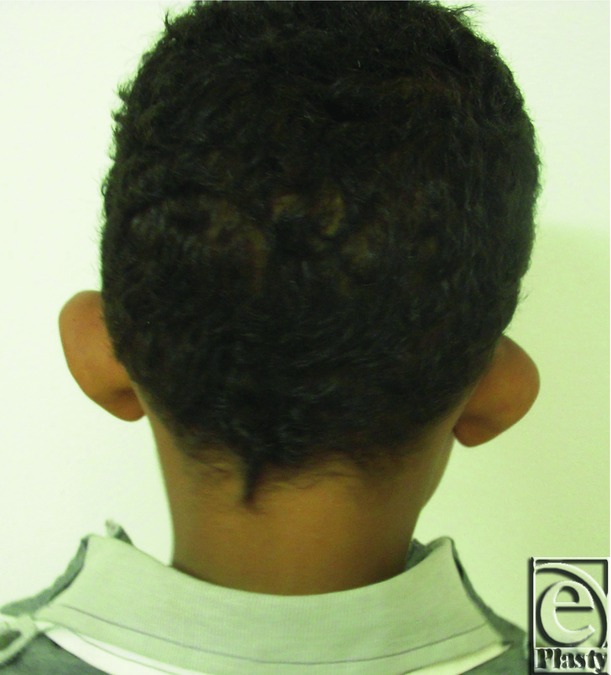
Preoperative posterior view of both ears in a 7-year-old male patient.

**Figure 14:**
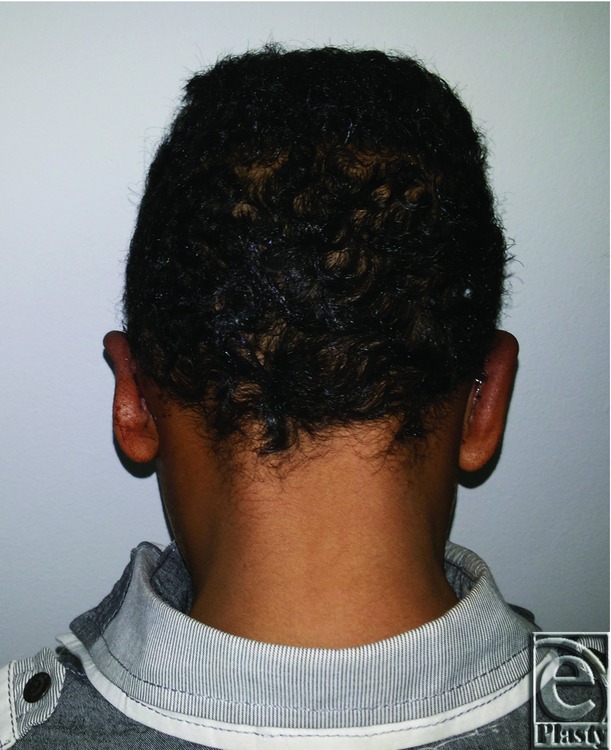
Postoperative posterior view of both ears for the same patient in Figure 13.

**Figure 15:**
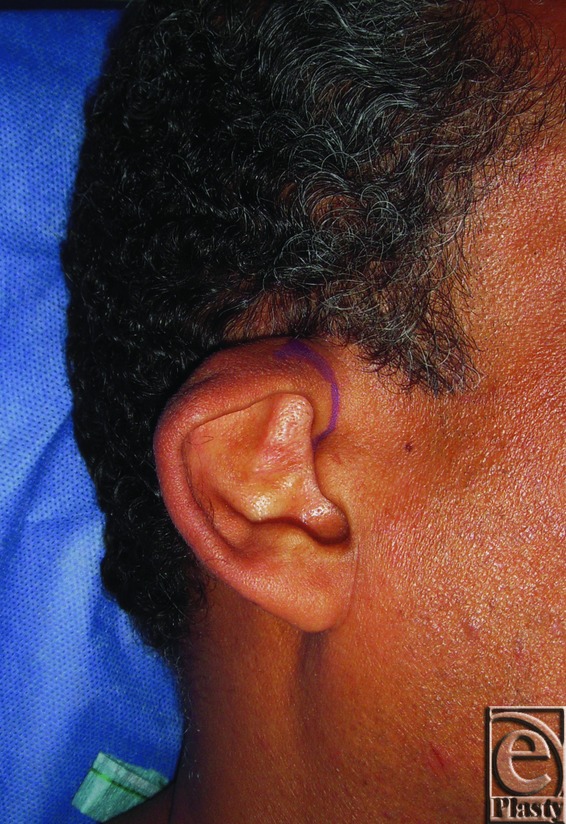
Constricted right ear in a 55-year-old male patient.

**Figure 16:**
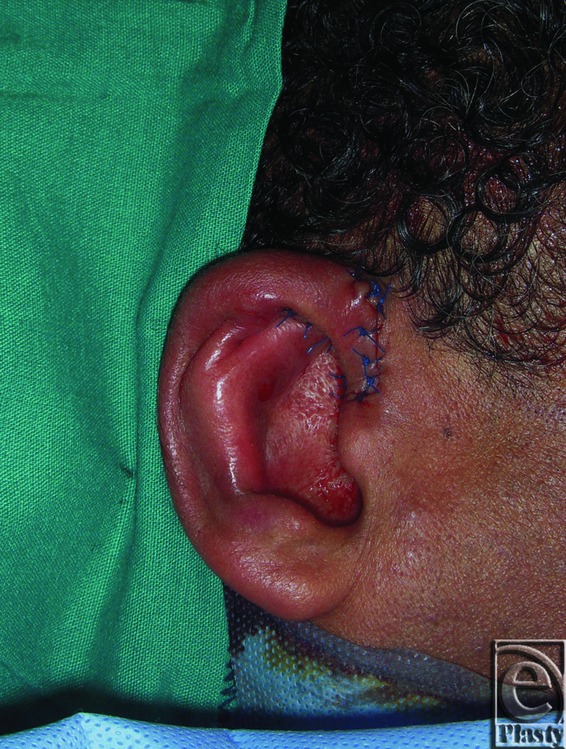
Postoperative photograph for the same ear in Figure 15.

**Figure 17:**
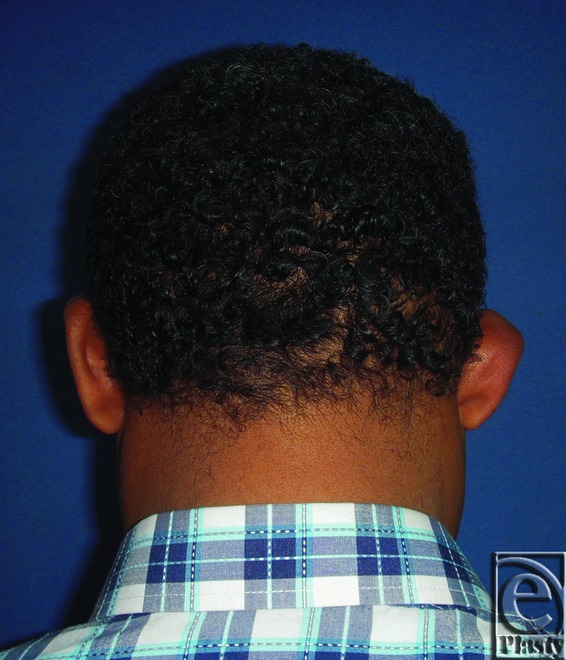
Preoperative posterior view of both ears in a 55-year-old male patient.

**Figure 18:**
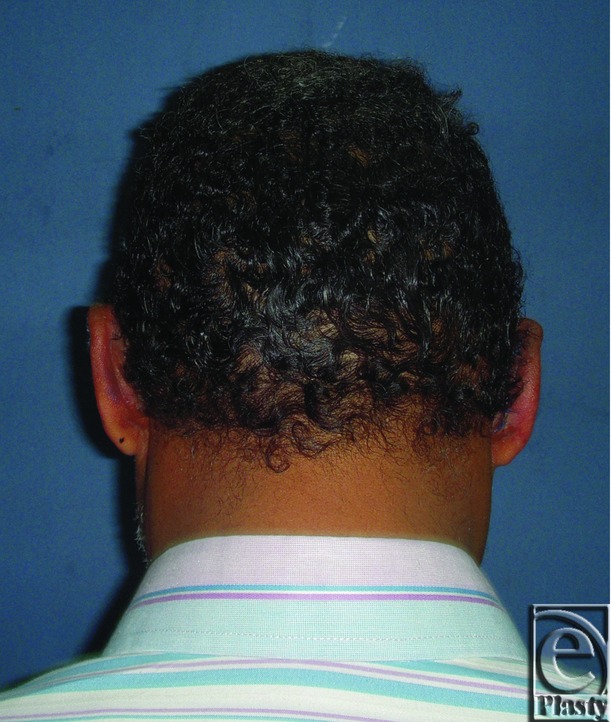
Postoperative posterior view of both ears for the same patient in Figure 17.

**Figure 19:**
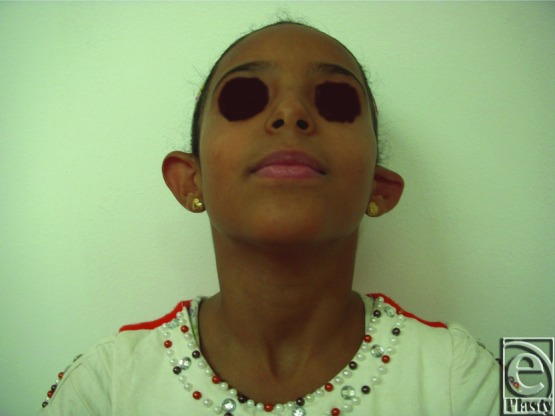
Preoperative anterior view of both ears in a 9-year-old female patient.

**Figure 20:**
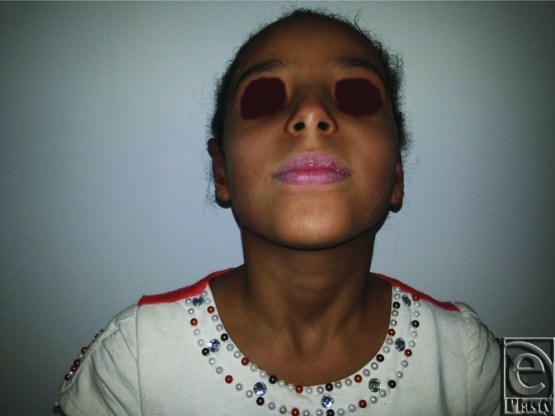
Postoperative anterior view of both ears for the same patient in Figure 19.

**Figure 21:**
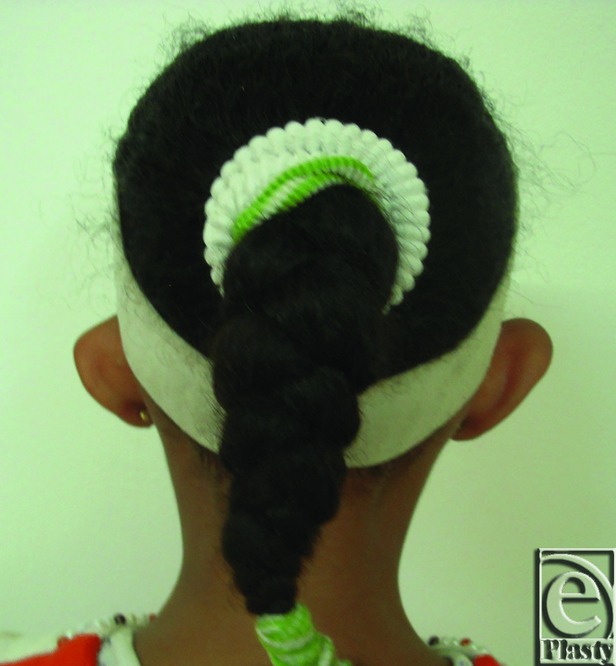
Preoperative posterior view of both ears in a 9-year-old female patient.

**Figure 22:**
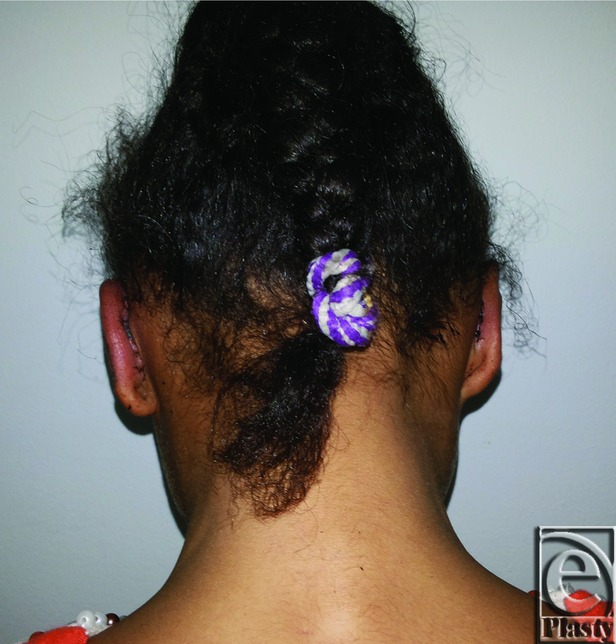
Postoperative posterior view of both ears for the same patient in Figure 21.

**Table 1:** The distribution of the age, gender, uni/bilateral, Tanzer type, contralateral ear deformity, and the procedures done on the contralateral ear in each patient

Patient number	Age	Gender	Uni/Bilateral	Tanzer type	Contralateral ear deformity	Procedures done on the contralateral ear
1	55 y	Male	Unilateral	IIA	Normal	Conchal cartilage graft harvested only
2	45 y	Male	Unilateral	IIA	Protruded	+ Set back
3	27 y	Male	Unilateral	IIA	Protruded	+ Creation of antihelical fold
4	10 y	Male	Unilateral	IIA	Normal	Conchal cartilage graft harvested only
5	10 y	Male	Unilateral	IIA	Protruded	+ Set back
6	9 y	Female	Unilateral	IIA	Protruded	+ Set back
7	9 y	Male	Unilateral	IIA	Protruded	+ Creation of antihelical fold
8	8 y	Male	Unilateral	IIA	Protruded	+ Set back
9	8 y	Male	Unilateral	IIA	Protruded	+ Set back
10	7 y	Male	Unilateral	IIA	Protruded	+ Set back
11	6 y	Female	Unilateral	IIA	Protruded	+ Creation of antihelical fold
12	6 y	Male	Unilateral	IIA	Protruded	+ Set back

**Table 2:** Analyzing the vertical height of the constricted and contralateral ears and the size difference between both ears pre- and postoperatively

Variables	Constricted ear	Contralateral ear	% of change	*t*	*P*
Preoperative vertical height	5.1 ± 0.4	5.7 ± 0.4	11.7	36	.000
					HS
Postoperative vertical height	5.8 ± 0.3	5.9 ± 0.4	2.5	1.9	.11
					NS

Abbreviations: HS, highly significant; NS, nonsignificant.

**Table 3:** The mean vertical height of the constricted ear pre- and postoperatively

Variables	Pre	Post	% of change	*t*	*P*
Vertical height of constricted ear	5.1 ± 0.4	5.8 ± 0.3	12.2	25	.000
					HS

Abbreviation: HS, highly significant.

**Table 4:** The mean pre- and postoperative vertical height of the contralateral ears

Variables	Pre	Post	% of change	*t*	*P*
Vertical height of contralateral ear	5.7 ± 0.4	5.9 ± 0.4	4	5	.000
					HS

Abbreviation: HS, highly significant.

**Table 5:** The mean change between the pre- and postoperative vertical height of the constricted and contralateral ears

Variables	Constricted ear	Contralateral ear	% of change	*t*	*P*
**Pre/post**	5.9 ± 1	1.9 ± 0.5	86	13	.000
					HS

Abbreviation: HS, highly significant.
